# Anticipating the course of an action: evidence from corticospinal excitability

**DOI:** 10.1186/1471-2202-14-91

**Published:** 2013-08-28

**Authors:** Mattia Marangon, Giulia Bucchioni, Stefano Massacesi, Umberto Castiello

**Affiliations:** 1 Department of General Psychology, University of Padova, Via Venezia, Padova 8-35131, Italy; 2 Laboratoire de Neuroscience Functionelles et Pathologies, UFR de Médicine, Université de Picardie Jules Verne, Amiens, France

**Keywords:** Transcranial magnetic stimulation, Cortical excitability, Intracortical inhibition and facilitation, Motor evoked potentials, Action planning, Multi-step action

## Abstract

**Background:**

Anticipatory planning, the ability to anticipate future perceptual-motor demands of a goal-oriented action sequence, is essential for flexible, purposeful behavior. Once an action goal has been defined, movement details necessary to achieve that goal can be selected. Here, we investigate if anticipatory planning takes place even when multi-step actions are being carried out. How, we may ask, are the cerebral circuits involved in movement selection influenced by anticipated object-center task demands? Transcranial magnetic stimulation (TMS) was used to investigate how changes in corticospinal excitability (CSE) are dependent on anticipated task variables of intended future actions. Specifically, single- and paired-pulse TMS was used to evaluate corticospinal excitability during the action selection phase preparatory to grasp execution.

**Results:**

We found that during the premovement phase, there is an object- and muscle-specific modulation in the intrinsic hand muscle that will be used during a forthcoming grasping action. Depending on whether the participants were instructed to perform a single- or double-step movement sequence, modulation of the corticospinal output to the appropriate hand muscles was dependent on what object was to be grasped and what type of movement was being prepared. No modulation in excitability was observed during one-step movements.

**Conclusions:**

Anticipation of intended task demands plays an important role in controlling multi- step actions during which ongoing behavior may need to be adjusted. This finding supports the notion that the cortico-cortical mechanism involving movement planning is specific for an object’s properties as well as for the goal of the movement sequence.

## Background

One of the most remarkable abilities that allows humans to interact with their environment is skilled object manipulation. Knowledge about an object’s properties, together with information regarding the current state of the system, including the initial configuration of the motor apparatus and where objects are located, are necessary if an individual is to flexibly and purposefully adapt motor output with reference to an action goal.

Neurophysiological evidence indicates that computations regarding prehensile actions occur within a lateral parietofrontal circuit involving the inferior intraparietal (area AIP in the macaque) and the ventral premotor cortex (area F5 in the macaque) [1-6]. It is generally agreed that area F5 plays a primary role in selecting the most appropriate hand and finger configuration depending on the object’s intrinsic properties provided by AIP to which it is reciprocally connected, thereby activating a motor representation of the object [7-10].

Consistent with observations in monkeys, activation of human AIP (hAIP) area is observed when objects are grasped under visual guidance, but it also seems to be tuned to the type of grasp to be adopted during movement planning [11]. The ventral premotor cortex (PMv) as well as the dorsal aspect of the PM cortex appear to be involved in controlling the more complex or goal-related aspects of reach-to-grasp actions [12]. The primary motor cortex encodes basic motor control parameters such as movement direction and force [13]. Recent functional magnetic resonance imaging (fMRI) studies nevertheless suggest that activity in the motor cortex (M1) is modulated by the level of congruence between the type of grasp expected and the size of the stimulus [11].

The boundaries of this parieto-premotor network have recently been extended by the discovery of the superior parieto-occipital cortex (human SPOC) which is a critical node for skilled grasping movements. Those studies suggest that the SPOC area, previously implicated in reaching, is also specialized for the arm transport phase during grasping movements [14,15].

Transcranial magnetic stimulation (TMS) studies have moreover verified the relevance of the parieto-frontal network for manual grasping [16]. As single TMS pulses have been found to briefly (50–150 ms) interfere with ongoing brain activity at the stimulated site, they can be viewed as producing a short-lasting, reversible functional lesions of that area. Behavioural changes induced by TMS can therefore potentially reveal information regarding the role played by the portion of the cortex that undergoes stimulation during a given task [17].

These studies confirmed that an object’s properties are encoded as a gradient along the human AIP-PMv-M1 axis, with the object first being represented as visual attributes and then in terms of an appropriate grasp [16,18-21]. Delivery of TMS over one of these regions in a specific time window interferes with the sensory-motor control of grasping [18].

A number of different neurophysiologic measures of cortical excitability, including motor threshold, motor evoked potential (MEP), and a silent period duration, can moreover be derived from single-pulse TMS. The MEP amplitude is the summed result of direct and indirect (trans-synaptic) excitation of a pool of corticospinal neuronal elements beneath the TMS coil which provides an immediate measure of cortical excitability at any given moment for any given condition (i.e. context or task dependent) [17,22]. Like single-pulse TMS, paired-pulse TMS has been shown to be a sensitive method to measure cortico-cortical interactions, and has been used successfully in experiments assessing the excitability of circuits intrinsic to the cerebral cortex itself [23,24].

More specifically, paired-pulses TMS were delivered in close succession to the same cortical region through a single stimulation coil. The first (conditioning) stimulus (CS) modifies the response to the second (test) stimulus (TS). The effects of the paired-pulse technique depend on the intensity of the CS, the interval between the stimuli (ISI), and the intensity of the TS. The intensities of the CS and TS influence the effects as distinct circuits are recruited by different stimulation intensities [25].

It has been found that paired-pulse TMS applied over the primary motor cortex with an interstimulus interval of about 1.2 or 2.5 ms determines MEP facilitation in the hand muscles involved in subsequent movements. Also known as short intracortical facilitation (SICF), this effect seems to be partially due to interactions between I waves and is thought to take place in the motor cortex or upstream from the cortico-spinal neuron [23-25]. Cattaneo and colleagues demonstrated that there is an enhancement in the excitability of those inputs to the corticospinal neurons projecting from the motor cortex to the specific muscles to be used during grasping actions at least 600 ms before the movement itself takes place. Those changes are object and muscle-specific, and the degree of modulation in the inputs is correlated with the pattern of muscular activity later used to grasp an object. Those authors speculated that this modulation probably reflects inputs from PMv to M1 that were activated by the sight of an object [23]. In a more recent study, that same group of researchers reported that changes in intracortical excitability were found to occur only if the object was specified by simultaneous visual input at the moment the grasp was initiated. But when subjects had to remember the object, even for just 1 s, a different neural network seemed to be used for motor preparation, suggesting that those circuits contribute to action selection only when immediate sensory information specifies what action is to take place [24].

Those studies focused on single-step actions whose immediate goal was to grasp an object. Until now, action goals have typically been investigated using single reaching or grasping acts [12]. During daily activities, however, hand-object interactions are not limited to single-step movements but tend to be multi-step sequences driven by a final goal (e.g. grasping a glass to take a drink of water).

We designed a paradigm to investigate if object and muscle-specific corticospinal excitability (CSE) is detectable when grasping is not the immediate but only the final goal of a more complex motor sequence. The paradigm was employed with the intent of shedding light on these interactions and of answering the following questions: Are aspects of a grasping motor plan detectable during an early premovement stage even when the immediate sensory information calls for a different action? Does the presence of a ‘first’ action delay the emergence of grasp-related CSE?

Tasks were thus designed to assess MEP modulation and context dependent aspects during the preparation of one- and two-step actions. In one condition, a single-step movement was connected to an immediate goal (to press a button centrally located); in another, a double-step movement driven by a final goal was carried out (the participant first pushed a button and then grasped one of variously sized objects located either to the left or to the right of the central button). This protocol made it possible to evaluate the role played by CSE in controlling multistep actions during which ongoing behavior may need to be adjusted in anticipation of forthcoming task demands.

We hypothesized that if higher pre-movement facilitation is specifically connected to the intended grasp, this would indicate that grasp-related premotor-motor (PMC-M1) cortex connectivity is already present during the preparation of action sequences. Modulation of MEP amplitude would thus predict the muscle activity that will be used to achieve the final goal of an action sequence. Consistent with previous findings [23,24], we did not expect to detect any changes in CSE for single-step actions linked to non action-relevant objects.

## Results

Peak-to-peak amplitudes of MEPs were measured for all of the experimental conditions (the various sized and located objects, the number of movements, and the TMS delivery times). Pre-movement facilitation was measured using single- and paired-pulse (ISI 2.5 ms) TMS. We calculated the ratio between the average amplitude of MEPs evoked by paired-pulse TMS and that of MEPs evoked by single-pulse TMS for the corresponding condition. This was calculated for every participant, for each muscle, and for every stimulus and TMS delivery time condition. A ratio value > 1 indicated increased cortico-spinal excitability, while values < 1 indicated a decreased one. A preliminary analysis was performed to verify differences depending on the location of the stimulus for each of the dependent measures considered. Those analyses indicated that there were no significant differences associated to stimuli located either to the right or to the left of the button (*p*_s_ > .05). That variable was thus collapsed during the main analyses. Data for each muscle were analyzed using two separate repeated measures ANOVAs with type of stimulation (single, paired pulse), type of movement (one-step, two-steps), stimulus size (small, large), and TMS delivery timing (200 ms, 800 ms) as within-subjects factors. Additional repeated measures ANOVA tests were performed on MEP facilitation ratios with types of movement, stimulus size and TMS delivery timing as within-subjects factors. Post-hoc pairwise comparisons were carried out using t-tests, and Bonferroni corrections were applied when necessary. A significance threshold of *p* < 0.05 was set for all statistical tests. Sphericity of the data was verified prior to performing statistical analysis (Mauchly’s test, *p* [0.05]) [26]. The data of three of the enrolled participants were removed from analysis: one because of equipment failure and the other two because some data concerning the experimental conditions were missing.

### First dorsal interosseus muscle (fdi)

#### ***TMS - Single Pulse (SP)***

**Figure 1:**
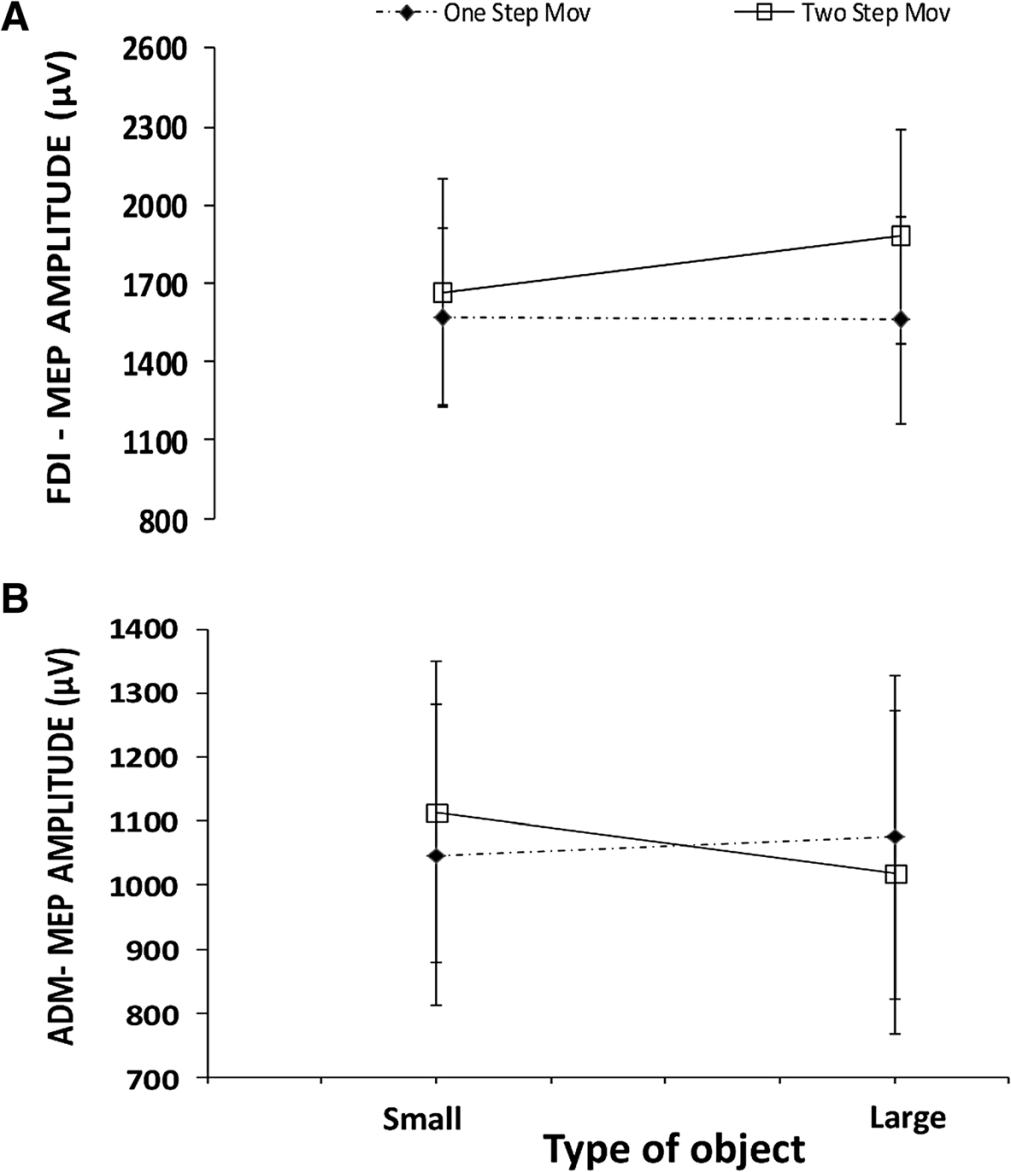
**Single-pulse TMS.** The effects of object sizes and types of movement on M1 excitability elicited by single-pulse TMS recorded in 17 participants. Panels **(A)** and **(B)** show changes in MEP amplitude recorded from the right first dorsal interosseous (FDI) **(A)** or the right abductor digiti minimi (ADM) **(B)** muscles. Errors bars indicate S.E.M.

We found a significant interaction between stimulus size and the type of movement for the FDI muscle (F (1,16) = 6.44, *p* = .02). MEP amplitude was suppressed for the double-step movements when the action goal was to grasp the small rather than the large stimulus (*t*(16) = 2.53, *p* = .03; Figure 1A). This difference was not present when the participants performed single-step actions.

#### ***TMS - Paired Pulse (PP)***

The MEPs elicited by paired-pulse TMS (ISI 2.5 ms) revealed a main effect of stimulus size for FDI (F (1,16) = 7.44, *p* = .01). MEP amplitude for the FDI muscle was higher for the small than for the large object (2272.80 μV vs. 2070.29 μV).

**Figure 2:**
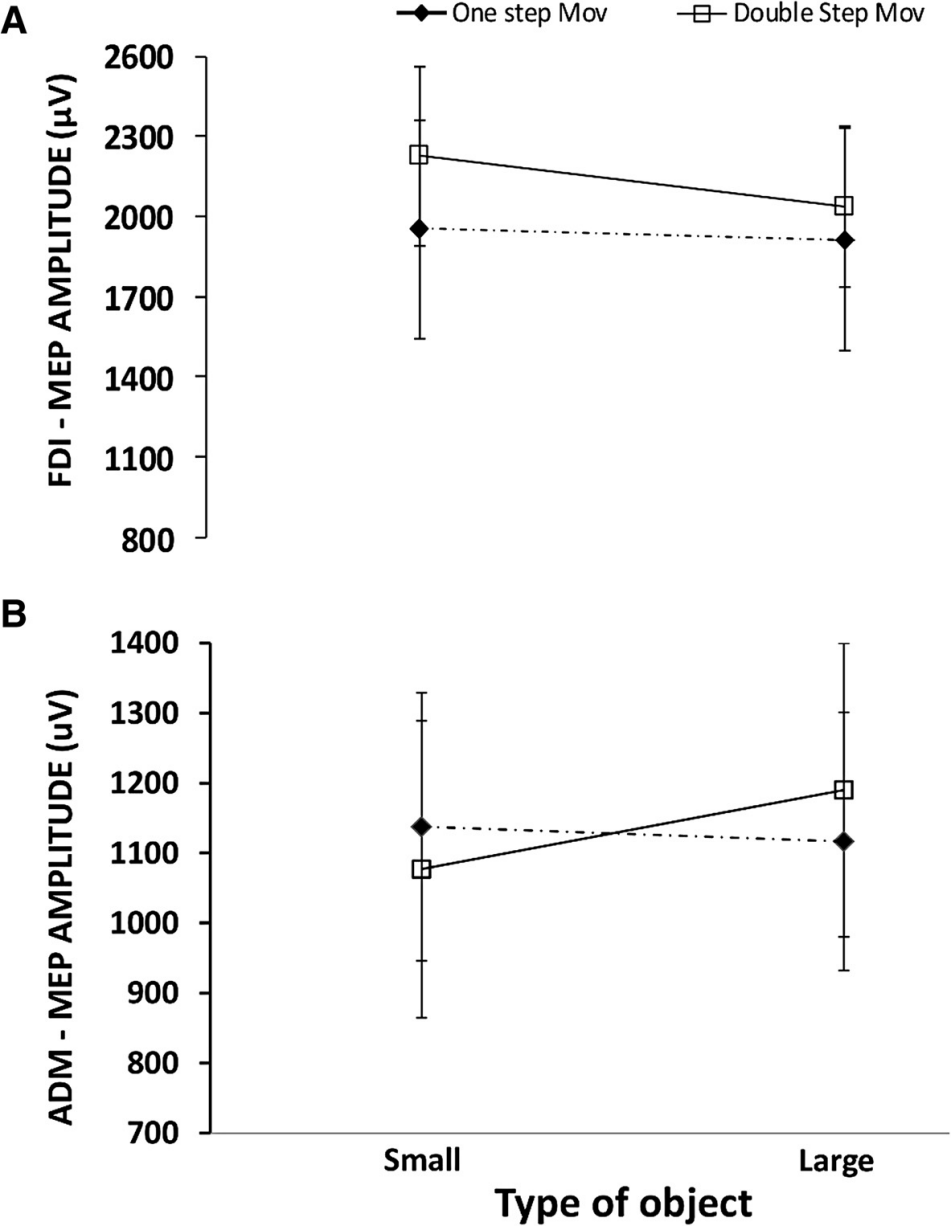
**Paired-pulse TMS.** Comparisons of MEP amplitude (n = 17) evoked by paired-pulse TMS (ISI = 2.5 ms) from the FDI. **(A)** and ADM **(B)** muscles. MEPs for TMS delivery timings (200 ms, 800 ms) were collapsed. Error bars represent the S.E.M.

MEP amplitude showed no significant variations for the FDI muscle in connection to the type of movement or stimulus size (F (1,16) = .53, *p* = .48) (Figure 2A).

### Abductor digiti minimi muscle (adm)

#### ***TMS - Single Pulse (SP)***

There was a significant main effect of stimulus size (F (1,16) = 7.74, *p* = .01) for the ADM muscle indicating that MEP amplitude was lower for the larger than for the smaller object (1016.50 μV vs 1055.50 μV). An interaction between the type of movement and the object size was detected (F (1,16) = 4.79, *p* = .04). MEP amplitude was significantly decreased for the larger with respect to the smaller object (*t*(16) = 2.18, *p* = .04 ) only when the participants were asked to perform double-step movements (Figure 1B).

#### ***TMS - Paired Pulse (PP)***

The MEPs elicited by paired-pulse TMS (ISI 2.5 ms) revealed a main effect of stimulus size for ADM (F (1,16) = 4.773, *p* = .04). MEP amplitude was more pronounced for the larger with respect to the smaller object (1037.90 μV vs. 995.60 μV). The interaction between the type of movement and the stimulus size was significant for the ADM muscle (F (1,16) = 8.575, *p* = .01; Figure 2). MEP amplitude for the ADM was higher for the larger with respect to the smaller object (*t*(16) = 2.92, *p* = .03*)* for the double-step movements. There was no difference across the stimuli (Figure 2B) for the single-step movements.

#### ***MEP facilitation ratio***

**Figure 3:**
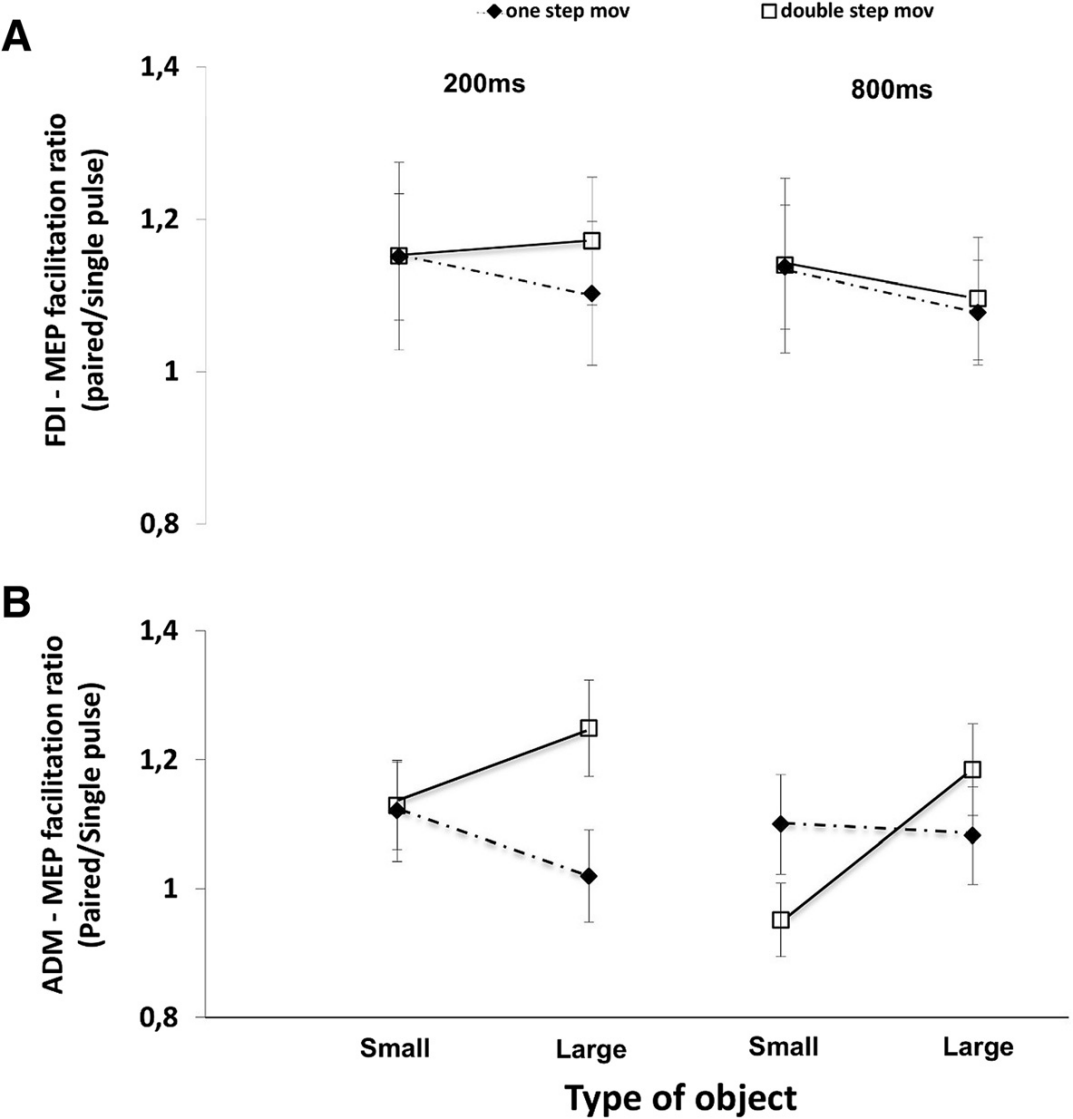
**MEP facilitation ratio.** MEP facilitation ratio (n = 17) (paired-pulse MEP amplitude at 2.5 ms interstimulus intervals (ISI) /single-pulse MEP) for TMS delivery times at two fixed intervals (200 ms and 800 ms) calculated from the time the pulses were delivered during grasping preparation. Panels **(A)** and **(B)** show changes in MEP amplitude recorded from the right first dorsal interosseous (FDI) **(A)** or the right abductor digiti minimi (ADM) **(B)** muscles. TMS was delivered after object presentation. Error bars represent the S.E.M.

We examined the contribution of single- and paired-pulse (ISI 2.5 ms) TMS as a measure of facilitation using paired-pulse TMS. We calculated the ratio between the average amplitude of MEP evoked by paired-pulse TMS and the average amplitude of MEP evoked by single-pulse TMS evoked for the corresponding condition. This was calculated for each participant as well as for each muscle, object, and TMS delivery time condition. The MEP facilitation ratio for the FDI muscle was not significantly different in connection to stimulus size and type of movement (Type of movement × Stimulus size, F (1,16) = .286, *p* = .60; Figure 3). The three way interaction - type of movement, TMS delivery time and stimulus size - was significant for the ADM muscle (F (1,16) = 4.780, *p* = .02; Figure 3). MEP facilitation was significant when the participants were instructed to perform double-step movements in order to grasp the larger with respect to the smaller object at the shorter TMS delivery time (*t*(16) = 2.18, *p* = .02). These results show that MEP modulation during grasp preparation is a predictor of the muscle activity that will be used during double-step grasping movements. This MEP facilitation ratio reflects a contrasting pattern of paired and single-pulse responses only when the participant is instructed is to reach and grasp an object. No significant effects were detected for the FDI muscle.

## Discussion

Single- or paired-pulse TMS was delivered at specific intervals during these experiments to investigate changes in the excitability of corticocortical inputs to the motor cortex as subjects prepared to grasp different sized objects in single- or two-step movements. The study was, in fact, designed to investigate how the goal of an action sequence and the information about the geometric features of the object-to-be-grasped affect motor programs.

Our results are consistent with observations from previous studies suggesting that excitatory inputs to M1 are modulated during motor preparation [23,24]. Those studies indicated that there were object- and muscle-specific changes in the cortical excitability of M1 immediately before a participant grasped an object. Those authors reported, in fact, that object-specific muscle activity differed when a participant was instructed to grasp a handle or a disc. MEP facilitation was, moreover, specific for the ADM muscle following paired-pulse TMS stimulation when an object was visually present throughout premovement selection period [23,24]*.*

Our results have confirmed these findings by showing that the relationship between MEP facilitation and premovement muscle activity preparation is affected by an object’s intrinsic features. There was, instead, no modulation of excitability when an object was present but the action goal did not entail grasping it. Our results take previous findings a step further because they indicate that MEPs are modulated not only when grasping an object is the immediate goal but even when it is the final objective of an action sequence. This signifies that the overall goal is embedded at the motor preparation stage and managed at the CSE level. More specifically, MEP facilitation was noted in the ADM muscle during motor preparation that was object and type-of-movement specific. Paired-pulse MEPs in the ADM muscle showed greater object specificity for grasping during double-step movements.

Interestingly, when the goal of a double-step action was grasping a small object, there was a decrease in MEPs amplitude for the FDI muscle when a single-pulse TMS was delivered and the effect remained constant during the interval between the TMS delivery times. By contrast, paired-pulse stimulation in the same muscle showed an object-specific (i.e., small vs. large) related MEP facilitation, but the differences were less marked than for the ADM muscle (Figure 2). Recordings from active muscles may have contributed to our failure to elicit facilitation in the FDI muscle at an ISI of 2.5 ms as MEP facilitation tends to be weaker in active muscles [27]. The absence of facilitation in these experiments could have been due to reduced excitability of motor cortical output cells or of interneurons, or of both. Cortico-cortical inputs from premotor areas can, moreover, inhibit the corticospinal neurons [28].

Our results suggest that the relationship between MEP facilitation and muscle activity is specific in conditions of sustained visual input and indicate that there is a clear distinction in preparatory movements depending on the immediate as well as the overall goals. A double-step movement preparation involves mechanisms associated with covert preparatory states and motor sequence planning. Actions are planned through a selection process during which multiple movements are chosen and placed in sequence, a process that is not immediately implied by the object’s intrinsic properties. Intended hand movements are initially encoded as object representations modulated by an action context, rather than as representations of a particular hand/finger configuration in purely motor terms.

As reflected in the excitability level of relevant muscle representations, the corticospinal system seems to be able to store an internal representation of motor outputs and can rapidly adapt its state to generate the most appropriate commands.

We were able to investigate modulation of inputs to M1 excitability during premotor preparation and disentangle effects stemming from different variables by dissociating the time of TMS delivery from object presentation and the grasping cue. Some authors have shown that task-related modulation of the paired-pulse MEP was abolished when stimulation was delivered 400 ms before the cue was given or when go signal was unpredictable [24]. Since a similar pattern was not noted during our experiments, we hypothesize that the parieto-frontal circuit can prepare and maintain grasp-related motor programs during a delay period.

Functional magnetic resonance imaging used to study predictive mechanisms of multi-step action planning in humans revealed a large network, including the dorsal premotor cortex (dPMC), the superior, parietal lobule (SPL) and along the IPS, that is active in response selection [29]. More recent findings have demonstrated that the entire network of brain areas implicated in response selection is also sensitive to intended task demands. Areas in both the parietal and frontal cortex increase their activation during preparation of multi-step, overall goal-directed actions [30]. These results suggest that movement planning involves internal representations of task demands that go beyond perceptual object information and that are formed well in advance of movement execution as part of a larger action sequence.

These cortical regions could support forward internal models that predict sensory consequences of a motor command slightly ahead of sensory feedback that accompanies movement. Internally guided motor programs are used when visual information and the goal of an action sequence are available to switch back and forth between different models of predictive control during grasping [31,32].

Our results suggest that this predictive internal representation operates in M1 by modulating corticospinal excitability. The advantage of this kind of anticipation is that it permits a rapid behaviour adjustment that would impossible if it depended on feedback alone. The individual optimizes his/her own performance by selecting lower level constraints which are context dependent facilitating successful completion of an action goal. The constraints that are chosen are measured and placed in a task-specific constraint hierarchy.

## Conclusions

Our results provide evidence that the level of CSE while reach-to-grasp movements are being prepared is determined by the intrinsic properties of the object and by the action goal.

The parieto-premotor network involved in preparing and maintaining grasp motor programs is also crucial in action selection during which context information specifies what actions will follow.

Although previously considered as a low-level motor output structure, the motor cortex is predominantly engaged in movement execution, and the fact that we can decode each particular hand movement from preparatory motor responses before action execution takes place suggests that it may play a more prominent role in movement planning processes.

## Methods

### Participants

Twenty healthy right-handed individuals (12 females and 8 males, mean age (± S.D.) 26.5 ± 5.58 years) who gave informed consent were recruited and participated in the study, which was approved by the Ethics Committee of the University of Padova and carried out in accordance with the Declaration of Helsinki (1964). Handedness was ascertained by means of the Edinburgh Handedness Questionnaire [33]. All the participants were healthy, had no history of neurological disorders, and had normal or corrected-to-normal visual acuity. All were screened for contraindications to TMS and were questioned about recent alcohol, caffeine and recreational drug use [34]. A verbal debriefing concerning the aims of the study was carried out at the end of experiment.

### Electromyographic and TMS recording

Monophasic TMS pulses were delivered using a 70-mm figure-of-eight coil connected to a Magstim BiStim^2^ (Magstim, Whitland, Dyfed, UK) placed over the left primary motor cortex (M1). The coil was placed tangentially on the scalp with the handle pointing backward and laterally at 45° to the sagittal plane, inducing a posterior-anterior current in the brain. TMS pulses were applied to the ‘hotspot’on the scalp where a MEP could evoke EMG recordings from two intrinsic hand muscles, namely the abductor digiti minimi (ADM) and the first dorsal interosseus (FDI). Once the ‘hotspot’ was localized, the site was marked with a red dot to ensure consistent coil positioning. The same experimenter was responsible for holding the coil in place for the duration of the experiment.

The intensity of TMS was adjusted to evoke a MEP of approximately 1 mV peak-to-peak in the relaxed right FDI and ADM. The hand motor area of left M1 was defined as the point where stimulation evoked the largest MEP from both muscles. The resting motor threshold (RMT), defined as the lowest stimulus intensity able to evoke MEPs greater than 50 μV in five out of ten trials, was assessed by holding the stimulation coil over both targeted muscles [35]. Surface EMG was recorded from these muscles through pairs of Ag-AgCl surface electrodes in a classical belly-tendon montage. Electrodes were connected to an isolate portable ExG input box linked to the main EMG amplifier for signal transmission via twin fiber optic cable (Professional BrainAmp ExG MR; Brain Products, Munich, Germany). EMG signals were amplified 2000 times, band-pass filtered (20 Hz – 2 KHz) and stored on a PC for off-line analysis. EMG recordings were started 100 ms before the magnetic pulse in order to control for the absence of muscular pre-activation in each trial. Trials in which EMG activity was greater than 100 μV within the 100 ms window preceding TMS pulse were discarded. Participants wore a tightly fitting bathing cap upon which the scalp positions for stimulation were marked. The coil was held by hand, and its position, with respect to the marks, was checked continuously. We chose to hold the coil by hand to easily compensate for small movements of the participant’s head during data collection. Held by hand or by a special holder, the coil has been found to induce a comparable MEP amplitude variability [36]. The stimuli were either single-pulse (130% RMT) or paired-pulse (130% and 90% RMT for the first and second stimulus, respectively). Paired-pulse TMS protocols have been used to study intracortical neural circuits in M1. Short-interval intracortical facilitation (SICF), known as facilitatory I-wave interaction, is elicited by a suprathreshold first stimulus (S1) and a subthreshold second stimulus (S2) [37-40] or two near-threshold stimuli [36] at very short ISIs. MEP facilitation is observed at three distinct phases at ISIs of 1.1–1.5 ms, 2.3–3.0 ms, and 4.1–5.0 ms in both arm and leg representations. The interstimulus interval (ISI) for paired-pulse TMS was 2.5 ms, which had been found to show object-specific muscle facilitation [23,24].

### Experimental stimuli

Four different stimuli were presented in random order: (i) a small cube (2 cm), (ii) a small sphere (2 cm), (iii) a large cube (8 cm); and (iv) a large sphere (8 cm). These objects were chosen because they are associated to different muscle groups during hand preshaping. Hand muscle activity was recorded from the FDI and the ADM muscles.

### Experimental procedure

**Figure 4:**
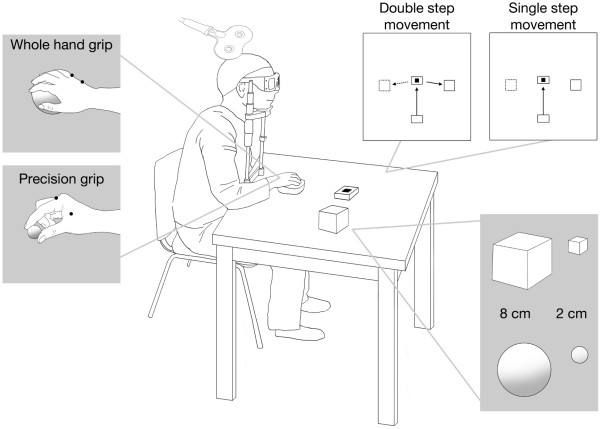
**Experimental setting.** Participants were seated in dimly lit room pressing with their right-hand on a home pad. They wore shutter glasses which could be switched from total blackness to transparent in order to permit selective vision of objects. Four objects were presented (2 large and 2 small). Participants were asked to perform one- or two-step movement after an auditory cue (a “go” signal), which was given 1200 ms after the shutter was switched to the transparent status. Only in the two-step trials were the participants instructed to grasp one of the objects. In the one-step trials the participants had to press a button in front of them in the presence of a non-action-related-object.

Participants were seated in a dimly lit room. They were instructed to position their right hand on a homemade hand support on the table (Figure 4). This starting position was the same for all conditions. The distance between the starting position of the hand and the nearest edge of a centrally located press button was 25 cm (Figure 4). Participants were asked to perform a one-step movement, i.e. to reach towards and press the button centrally located in front of them or a double-step movement, i.e. to reach out, to press the centrally located button and then to reach out and grasp one of the four objects positioned either to the left or to the right of the press button. The objects were either small, implying the use of a precision grip (i.e., the opposition of the thumb and the index finger), or large implying the use of a whole hand grip (i.e., the opposition of the thumb with all fingers) (Figure 4). Even during the ‘one-step movement’ one of the four objects was located either to the right or to the left of the central button (but the participants were not expected to grasp any of these).

The participants were instructed to carry out movements in a natural manner and they were not encouraged to rush. In order to control for the objects’ visibility, the participants were instructed to wear shutter lenses that could be switched from totally black to transparent during the experiment. The participants were asked not to initiate their movement until they received an auditory (‘go’ signal; Hz 400 Duration: 200 ms) cue. There was a fixed interval (1200 ms; ± 10% jitter) between visual presentation of the stimuli and the time the ‘go’ signal was given. TMS pulses were delivered randomly 200 and 800 ms after the objects were presented in 85% of the trials. To increase the unpredictability of the ‘go’ signal, no TMS was delivered in 15% of these trials. The two TMS delivery timings were selected on the basis of previous literature findings [23,24]. The shutter lenses remained opened for 3 s at the beginning of each trial to allow visual guidance of the movement. When the participant’s hand came back to the starting position, the shutter lens device was closed and remained closed until the next trial was begun. The inter-trial interval was 8 s long. The objects as well as the TMS conditions (single or paired-pulse) were presented in random order. The session consisted of four blocks of 36 trials; a total of 144 trials resulting from the combinations of four objects (as explained above), two positions (left or right of the central button), two types of movement (one or double-step) and two TMS delivery times (200 or 800 ms). Each participant took part in a mock training made up of 15 trials held at the beginning of the session to give them the opportunity to familiarize themselves with the tasks. During the practice session the participants were given information about premovement EMG and how they were expected to carry out the tasks.

## Competing interests

The authors have no disclosure of financial interests or potential conflict of interests to make.

## Authors’ contributions

MM designed the study, carried out the experiments, analyzed the data, performed the statistical analysis, and drafted the manuscript. GB coordinated study activities and carried out the experiments. MS was involved in the study’s technical aspects. UC designed the study, coordinated study activities, and drafted the manuscript. All the authors read and approved the final version of the manuscript.
